# Upregulation of activin-B and follistatin in pulmonary fibrosis – a translational study using human biopsies and a specific inhibitor in mouse fibrosis models

**DOI:** 10.1186/1471-2466-14-170

**Published:** 2014-11-01

**Authors:** Marjukka Myllärniemi, Jussi Tikkanen, Juha J Hulmi, Arja Pasternack, Eva Sutinen, Mikko Rönty, Outi Leppäranta, Hongqiang Ma, Olli Ritvos, Katri Koli

**Affiliations:** Department of Medicine, Division of Pulmonary Medicine, University of Helsinki and Helsinki University Central Hospital, PO Box 63, FI-00014 Helsinki, Finland; Transplantation Laboratory, Haartman Institute, University of Helsinki, PO Box 21, FI-00014 Helsinki, Finland; Department of Biology of Physical Activity, University of Jyväskylä, PO Box 35, Jyväskylä, FI-40014 Finland; Department of Bacteriology and Immunology, Haartman Institute; University of Helsinki, PO Box 21, FI-00014 Helsinki, Finland; Department of Pathology, Haartman Institute, University of Helsinki, PO Box 21, FI-00014 Helsinki, Finland; Research Programs Unit, Translational Cancer Biology, University of Helsinki, PO Box 63, FI-00014 Helsinki, Finland; HUSLAB, Helsinki University Central Hospital, PO Box 400, FI-00029 HUS Helsinki, Finland; Institute of Dentistry, University of Helsinki, PO Box 64, FI-00014 Helsinki, Finland; Biomedicum Helsinki C405b, University of Helsinki, PO Box 63, FI-00014 Helsinki, Finland

**Keywords:** Activins, Follistatin, Idiopathic pulmonary fibrosis, Mouse fibrosis model

## Abstract

**Background:**

Activins are members of the TGF-ß superfamily of growth factors. First, we identified by expression array screening that activin-B and follistatin are upregulated in human idiopathic pulmonary fibrosis (IPF). Next, we wanted to clarify their specific role in lung fibrosis formation.

**Methods:**

We used specific antibodies for activin-A and -B subunits and follistatin to measure and localize their levels in idiopathic pulmonary fibrosis and control lung biopsies. To inhibit activin signaling, we used soluble activin type IIB receptor fused to the Fc portion of human IgG1 (sActRIIB-Fc) in two different mouse models of pulmonary fibrosis.

**Results:**

Activin-B and follistatin mRNA levels were elevated in the human IPF lung. Immunoreactivity to activin-A, -B and follistatin localized predominantly to the hyperplastic, activated alveolar epithelium, but was also seen in inflammatory cells. Mice treated with sActRIIB-Fc showed increased skeletal muscle mass and a clear reduction in alveolar cell counts in bronchoalveolar lavage fluid, but no significant antifibrotic effect in the lung was observed.

**Conclusions:**

The upregulation of activin-B and follistatin in IPF is a novel finding. Our results indicate that activin inhibition is not an efficient tool for antifibrotic therapy, but could be useful in reducing alveolar cellular response to injury. Activin-B and follistatin levels may be useful as biomarkers of IPF.

## Background

Idiopathic pulmonary fibrosis (IPF) is a disease with a prognosis worse than many cancers [1]. Promising advances in the treatment of IPF have emerged recently [2–4]. The pathobiological alterations in IPF have been related to injury-induced epithelial cell activation and enforced transforming growth factor (TGF)-ß signaling. The clinical course of IPF is unpredictable; some patients develop acute worsening of the disease with no identifiable cause, i.e. acute exacerbations, where the mortality can be up to 70% [5]. The histopathological finding in IPF exacerbation is diffuse alveolar damage superimposed on the typical IPF histopathology of usual interstitial pneumonia (UIP) [6].

Activins belong to the TGF-ß superfamily of growth factors and are composed of two inhibin ß subunits. They signal mainly through activin type I and II transmembrane serine/threonine kinase receptors (ActRI and II) inducing Smad signaling pathways [7]. Both activin-A and -B signal through either ActRII or ActRIIB, as type II receptor and a common type I receptor known as ActRIB, or activin-like kinase 4 (ALK-4) to activate the Smad2/3 pathway. However, Smad signaling activation by activin-A and -B is not identical in target cells as activin-B, contrary to activin-A, also uses the ActRIC or ALK-7 type I receptor for Smad2/3 activation [8]. Activin-B can also act as a bone morphogenetic protein (BMP)-like factor by inducing Smad1/5/8 phosphorylation via type I BMP receptor BMPRIA or ALK-3 [9]. It is therefore important to distinguish between the relative roles of these two activins and their inhibitors in different tissue and disease entity contexts.

Activins were initially identified for their role in regulating reproductive processes. Later they were found to be involved in the regulation of inflammatory and tissue repair processes [10]. In the lung, increased activin levels are observed in antigen-induced airway inflammation [11], they are also associated with acute injury [12]. Activin-A immunoreactivity has been shown to increase in IPF and acute lung injury autopsy specimens [13]; activins have also been shown to associate with lung fibroblast proliferation and differentiation [14]. Adenoviral expression of activin-A in mouse lung epithelium leads to respiratory pathology similar to human acute respiratory distress syndrome involving inflammation, fibrosis, and tissue remodeling [12]. Follistatin is a natural activin-blocking molecule that is highly expressed in the ovary but is also found in a number of extragonadal tissues; its role is likely that of a natural quenching molecule that limits activin effects at the tissue level. Follistatin has been shown to suppress bleomycin-induced lung fibrosis and carbon tetrachloride-induced liver fibrosis in experimental mouse models [15, 16]. The contribution of the activin/follistatin system in the development or progression of IPF is not well known. Functional studies on lung fibrosis or localization of activin-B in the lung have not been published.

This study was designed to screen for potential novel indicators of TGF-ß signaling in the human IPF lung. We found that activin-B and follistatin mRNA were upregulated in the IPF lung. We also identified an increase in the immunoreactivity of activin-A, -B and follistatin in the pathological, hyperplastic epithelium in the human IPF lung. Activin neutralization by using a soluble ActRIIB receptor fused to the Fc portion of human IgG1 [12, 17] reduced bronchoalveolar lavage fluid (BALF) cellular responses to injury in two different mouse models but failed to show any significant antifibrotic effects.

## Methods

### Human tissue samples

An approval for collecting tissue for research purposes from diagnostic clinical samples was received from the Helsinki University Hospital Ethics Board (HUS 426/13/03/01/09). All patients that participated to the study gave written informed consent except for the lung transplant donors that were deceased. A permission to use tissue samples from deceased patients was obtained from the national supervisory authority of welfare and health (3317/05.01.00.06/2011).

For RNA isolation and expression array analysis, four IPF and four control specimens were carefully chosen in order to minimize variability due to confounding factors. Each patient’s medical history, HRCT evaluation and clinical course of the disease indicated that they had classical IPF; this was confirmed with lung biopsy. Three IPF biopsies were collected from explanted lungs at the time of transplantation, one from a diagnostic biopsy procedure. The mean age of the IPF patients was 55 (range 49–57), and all patients were nonsmokers. Control specimens in the array analysis consisted of patients who underwent surgical lobectomy due to benign pulmonary tumor (hamartoma), showed normal pulmonary histology, and had normal lung function (all patients were nonsmokers, mean age 64, range 59–70).

For immunohistochemical analyses, samples from diagnostic biopsies or lung transplantation, or both, were obtained from the University of Helsinki, Department of Pathology. Clinical records and HRCT scans were evaluated from all the patients according to the current guidelines to ensure correct diagnosis. Control lung samples were biopsies or segments of healthy donor lung that was used for transplantation. All the IPF patients had typical, advanced UIP pattern in the biopsy of the explanted lung. All the IPF patients were male. Their mean forced vital capacity (FVC % from predicted) before the operation was 47, and the mean volume-adjusted diffusing capacity was 60.5. Mean patient age was 52 (range 40–59).

### Antibodies

Mouse monoclonal antibodies (mAbs) against human activin-A, activin-B and follistatin subunits (encoded by *INHBA*, *INHBB* and follistatin genes, respectively) were generated by AnshLabs LLC (Webster, TX) by using peptide-conjugates that contain peptide sequences of the C terminal portion of the mature region of each activin. The activin-reacting mAbs were initially selected against commercially available human recombinant activin-A and -B (R&D Systems, Minneapolis, MN) and internally produced chinese hamster ovary (CHO) cell derived human recombinant activins at AnshLabs (not shown). The mAb 18/26A (for activin-A/INHBA), mAb 12/9A (for activin-B/INHBB) and mAb 4/73C for follistatin were selected for immunohistochemistry based on their specificity in Western blots and specific reactivity towards granulosa cells in human ovarian sections (not shown).

### Immunohistochemistry

Paraffin-embedded tissue sections were deparaffinized in xylene and rehydrated in graded alcohol. Antigens were retrieved by heating the sections in 0.01 M citrate buffer (pH 6.0). For immunostaining, Novolink Polymer Detection System (Novocastra, Leica Biosystems Newcastle Ltd., Newcastle Upon Tyne, UK) was used according to the manufacturer’s protocol. The sections were exposed to the primary antibodies at room temperature for 1 h. The bound antibodies were visualized by DAB. The sections were counterstained with Mayer’s haematoxylin and mounted on glass slides.

### SDS-PAGE and immunoblotting

Snap-frozen and subsequently pulverized mouse lung was lysed on ice for 15 min in RIPA buffer (50 mM Tris-HCl, pH 7.4; 150 mM NaCl, 1 mM EDTA, 1% NP-40, 0.2% sodium deoxycholate) that contained protease inhibitors (Pierce, Rockford, IL). Protein concentrations were measured using a BCA protein assay kit (Pierce, Rockford, IL). Equal amounts of protein were separated by SDS-PAGE using 4-20% gradient Tris-glycine gels (Lonza, Basel, Switzerland) and transferred to nitrocellulose membranes (Bio-Rad, Hercules, CA) using a semi-dry blotting system (Bio-Rad). Membranes were blocked with 5% non-fat milk in TBS/0.05% Tween-20 to prevent non-specific binding of the antibodies. Next, they were incubated with anti-inhibin ß_B_ monoclonal antibody (46A/F) [18], and then with biotin-conjugated anti-mouse secondary antibody (DAKO, Glostrup, Denmark) in TBS/0.05% Tween-20 containing 5% bovine serum albumin at room temperature. After several washing steps, the final detection was performed using horseradish peroxidase-conjugated streptavidin and an enhanced chemiluminescence Western blotting detection system (Amersham, Freiburg, Germany). Analyses of protein band intensities were performed using the Scion Image analysis program (Scion Corporation).

### RNA isolation and quantitative RT-PCR

Total RNA was extracted from homogenized lung tissue samples with an RNeasy Mini Kit (Qiagen GmbH, Hilden, Germany) and reverse transcribed using iScript cDNA synthesis kit (Bio-Rad). The cDNAs were amplified using TaqMan Assays-on-Demand gene expression products (Applied Biosystems) and CFX96 Real-time PCR detection system (Bio-Rad). Control amplifications directly from RNA were performed in order to rule out DNA contamination. The relative gene expression differences were calculated with the comparative delta delta cycle threshold (ΔΔCT) method, and the results have been reported as mRNA expression levels normalized to the levels of a gene with a constant expression (TATA-binding protein).

### Expression PCR array

Pathway-specific PCR array (#PAHS-035; SABiosciences) was used to analyze mRNA expression levels of genes associated with the TGF-ß/BMP signaling pathway. Following the manufacturer’s instructions, reverse transcription was performed using DNase I treated RNA and RT2 First Strand Kit (SABiosciences) followed by PCR amplification using CFX384 real-time PCR detection system (Bio-Rad). Gene expression levels in control lung tissue (n = 4) were compared to the levels in IPF lung tissue (n = 4) using SABioscience PCR data analysis tools.

### sActRIIB-Fc production

The recombinant fusion protein containing the ectodomain of human ActRIIB fused to the Fc domain of human IgG1 (sActRIIB-Fc) was produced in-house as described previously [12, 17]. For the final production of the chimeric protein, CHO cells were transfected with the expression construct via lipofection (Fugene 6; Roche) and selected with puromycin (Sigma-Aldrich). During selection, cells were grown in DMEM supplemented with 2 mM L-glutamine, 100 μl/ml streptomycin, 100 IU/ml penicillin, and 10% fetal calf serum. For large-scale expression, cells were adapted to CD OptiCHO medium (Gibco) supplemented with 2 mM L-glutamine and grown in suspension in an orbital shaker. Cell culture supernatants were clarified by filtration through 0.22 μm membrane (Steritop, Millipore). 1 M NaCl, 5 mM imidazole, and Protease Inhibitor Cocktail (Thermo Scientific) were added into the clarified supernatants, and the solution was pumped through a HisTrap FF Crude column (GE Healthcare Life Sciences, Uppsala, Sweden) at 4°C. Protein was eluted with increasing imidazole concentrations, dialyzed against phosphate-buffered saline (PBS), and finally concentrated with Amicon Ultra concentrator (30 000 MWCO, Millipore).

### Mouse models of IPF

A permission to use experimental animals was received from the regional state administrative agency of Southern Finland (ESAVI/871/04.10.03/2012) and their ethical board. The reporting on animal experiments was done in accordance to the Animal Research: Reporting of In Vivo Experiments (ARRIVE) guidelines (http://www.nc3rs.org.uk/page.asp?id=1357). Male C57b6J mice were used for the experiments. The mice were exposed to either crocidolite asbestos fibers (0.5 mg/50 μl sterile PBS) or silicon dioxide (SiO_2_) (Sigma; 2.5 mg/50 μl sterile PBS) as described previously [19]. Two subsequent oropharyngeal aspiration doses were given on days 0 and 7 of the experiment to ensure even exposure of all animals. The mice were harvested on day 28 after exposure. sActRIIB-Fc was administered via i.p. injections at a dose of 5 mg/kg twice a week during the entire observation period. Knowing that sActRIIB-Fc increases body mass, the mice were weighed at each drug injection to monitor the drug effect [17, 20, 21]. At the time of harvest, mice were euthanized with carbon dioxide, after which BALF samples were collected by injecting 2×300 μl of sterile PBS to the trachea. The BALF cells were counted using Bio-Rad TC10 automated cell counter. Differential cell counts were obtained by microscopy of May-Grünwald-Giemsa-stained cytocentrifuge preparates: 150 μl BALF was loaded to cytospin chambers containing Superfrost Ultra Plus glass slides (Menzel GmbH & Co KG, Braunschweig, Germany) and centrifuged for 8 minutes, 500 rpm. The right lung was snap-frozen and subsequently pulverized for hydroxyproline and RNA and/or protein analysis. The left lung was fixed with 3% paraformaldehyde for histological analysis. For sActRIIB-Fc administration and analysis of possible alterations in body composition due to asbestos or silica exposure or both, hindlimb skeletal muscles and epididymal/retroperitoneal fat was prepared and weighed using an analytical scale. Histological evaluation was performed as described and validated previously [22].

### Statistical analyses

All comparisons were made using nonparametric tests with SPSS software (IBM). Multiple group comparisons were made using Kruskal-Wallis test, and two-group comparisons were made using Mann-Whitney test. If multiple comparisons were made between two groups, the Bonferroni correction was applied to *P* values. *P* values below 0.05 were considered statistically significant.

## Results

### Expression of activin-A, -B and follistatin in human IPF lung

**Figure 1:**
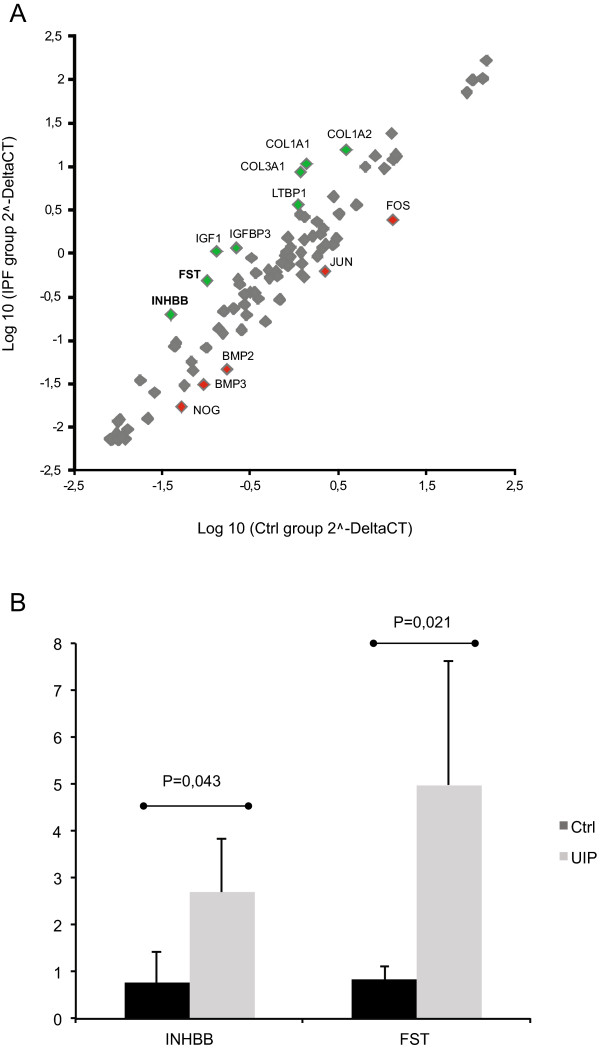
***INHBB*** **and** ***follistatin*** **mRNA expression is increased in IPF lung. A**. The mRNA expression levels of TGF-ß/BMP pathway genes in human idiopathic pulmonary fibrosis (IPF) lung (N = 4) and control lung (N = 4) were measured using a PCR array. In the scatter plot, IPF group is plotted against the control group. At least threefold differences in mRNA expression levels are indicated with green (upregulation) and red (downregulation) color. *INHBB* and *follistatin* mRNA expression was increased 3.8-fold and 3.6-fold, respectively. Col, collagen; LTBP1, latent TGF-ß binding protein; IGF, insulin-like growth factor; IGFBP, IGF binding protein; BMP, bone morphogenetic protein. **B**. *INHBB* and *FST* mRNA expression levels were confirmed by quantitative RT-PCR.

Total RNA was isolated from frozen human lung tissue samples from control subjects and IPF patients. A commercial PCR array was used to analyze the mRNA expression levels of the TGF-ß/BMP pathway genes (see Methods). As expected, the major fibrotic collagen genes *COL1A1* (p = 0.011)*, COL1A2* (p = 0.005) and *COL3A1* (p = 0.018) were significantly upregulated in the IPF patient lungs (Figure 1A). In addition, *LTBP-1* (p = 0.002), *IGF-1* (p = 0.011) and *IGFBP3* (p = 0.002) were found upregulated as previously described [23–25]. A trend for increased levels of *INHBB* mRNA (p = 0.088), which encodes activin-B subunit was observed. The mRNA expression of the activin inhibitor protein follistatin was also notably increased in the IPF lungs (p = 0.005). Upregulation of *INHBB* and *FST* mRNA levels were confirmed by quantitative RT-PCR (Figure 1B). The TGF-ß/BMP pathway genes *BMP2* (p = 0.072)*, BMP3* (p = 0.005)*, NOG* (p = 0.04)*, FOS* (p = 0.087) and *JUN* (p = 0.083) were downregulated in the IPF lungs.

**Figure 2:**
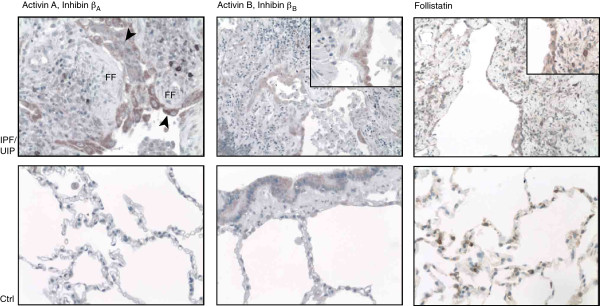
**Activin-A, -B and follistatin immunoreactivity in human usual interstitial pneumonia (UIP) lung tissue and control lung samples.** Activin-A immunoreactivity (upper panel, left) is observed in hyperplastic alveolar epithelial cells (arrowheads) overlying fibroblastic foci (FF). Occasionally, macrophage-appearing cells in the lung parenchyma stain positive. Activin-B immunoreactivity is similarly observed in areas of hyperplastic epithelial cells and, to a lesser extent, in parenchymal inflammatory cell infiltrates. Follistatin expression is observed at a high intensity in activated alveolar epithelial cells. Staining is also seen in lung bronchial epithelial cells (activins) and alveolar cells (follistatin), shown in the control (Ctrl) lung sections, lower panel.

Immunoreactivity for activin subunits was markedly increased in human IPF/UIP tissue when compared to controls, especially in the IPF/UIP lungs’ hyperplastic alveolar epithelial cells (Figure 2 and Table 1). Follistatin immunoreactivity was seen in the control and IPF lungs at high intensity. In the IPF/UIP tissue, high-intensity follistatin staining was seen in the activated alveolar epithelium and parenchymal macrophages. In control lungs, immunoreactivity was seen in macrophages or type-II-appearing cells or both. In control samples, immunoreactivity was observed in some alveolar macrophages and the bronchial epithelium for inhibin ß_A_ and follistatin, and for the inhibin ß_B_ subunit mainly in the bronchial epithelium. In IPF/UIP tissue, immunoreactivity for inhibin ß_A_ subunit was observed in the activated alveolar epithelial cells, macrophage-appearing cells in the lung parenchyma, and alveolar space, whereas fibroblastic foci in the lung parenchymal tissue were negative (Figure 2). Inhibin ß_B_ antibody showed immunoreactivity specifically in hyperplastic alveolar epithelial cells and bronchial epithelial cells. Macrophages remained mostly negative for inhibin ß_B_ in the IPF/UIP lung.

**Table 1:** **Localization of inhibin subunit antibody immunoreactivity in human control (Ctrl) and idiopathic pulmonary fibrosis (IPF) lung**

Patient	Inhibin ß _A_			Inhibin ß _B_			Follistatin		
Localization	(H)AEC	BE	MΦ	(H)AEC	BE	MΦ	(H)AEC	BE	MΦ
Ctrl1	-	-	++	-	-	-	-	+++	++
Ctrl2	-	+	+	-	+	+	-	-	++
Ctrl3	-	+	+	-	+	+	-	+++	+++
Ctrl4	-	-	-	-	+	+	-	++	++
Ctrl5	-	-	-	-	-	-	-	++	++
IPF/UIP1	++	++	++	+	+	-	+++	+++	+++
IPF/UIP2	+++	++	+++	+	+	-	++	++	++
IPF/UIP3	-	-	-	-	+	-	+++	+++	+++
IPF/UIP4	+++	+++	+++	+	-	-	++	++	+++
IPF/UIP5	++	++	++	+	++	-	+++	+++	+++

The histopathological finding of IPF is usual interstitial pneumonia (UIP). (H)AEC, (hyperplastic) alveolar epithelial cells; BE, bronchial epithelial cells; MΦ, macrophages.

### Expression of activin-A and -B in mouse models of pulmonary fibrosis

**Figure 3:**
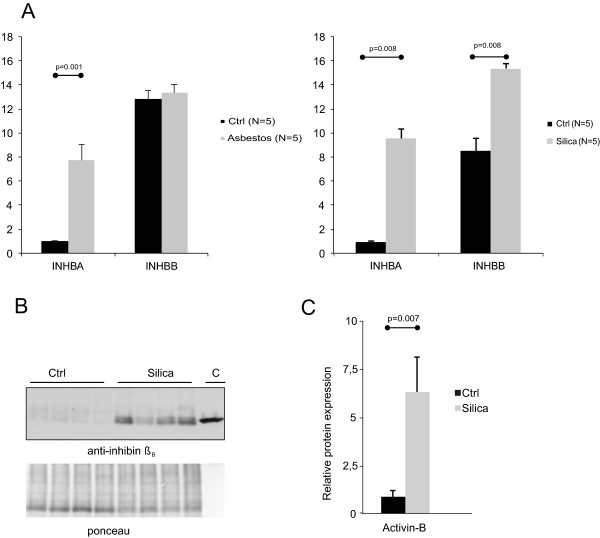
**Levels of Activin mRNA and protein in the mouse lung. A**. *Inhba* and *Inhbb* expression levels in asbestos (Asb)- and silica-exposed mice at 30 days after oropharyngeal aspiration. The expression levels are shown relative to the expression levels of *Inhba* in control-1. Control (Ctrl) animals are non-treated and non-exposed mice. **B**. Immunoblotting analyses of lung tissue lysates using anti-inhibin ß_B_ specific antibodies. The mobility of recombinant 25 kDa human activin-B (C, control) is shown on the right. Ponceau staining indicates equal loading. **C**. Quantification of inhibin ß_B_ band intensities.

Next, we analyzed the expression of activin subunits in two different mouse models of IPF, asbestos-induced fibrosis and silicon dioxide (silica)-induced fibrosis. In both models progressive fibrosis is observed, and the histopathological alterations resemble human IPF/UIP: patchy areas of fibrosis and inflammatory cell aggregates and areas of preserved normal lung architecture. In control mice, *Inhbb* mRNA was expressed at 7–10 fold higher levels than *Inhba* mRNA (Figure 3A). In both models, *Inhba* mRNA was markedly increased in the fibrotic lung tissue, whereas *Follistatin* levels remained essentially unaltered (Figure 3A). Interestingly, in the silica-induced fibrosis model also *Inhbb* mRNA levels were significantly elevated in the lung tissue (Figure 3A). The upregulation of activin-B in the silica-induced fibrosis model was confirmed at the protein level (Figure 3B and C).

### Neutralization of activins in mice by using sActRIIB-Fc

**Figure 4:**
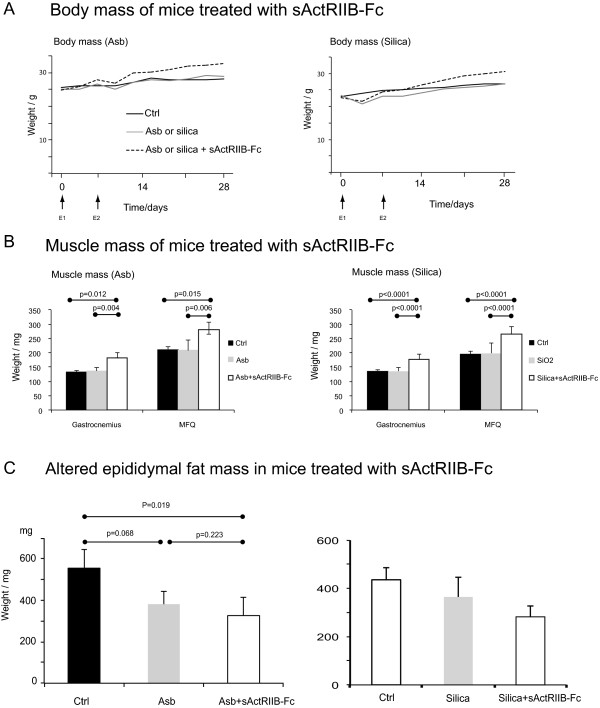
**Effect of activin inhibition to mouse body composition after particulate exposure. A**. Effect of activin inhibition using sActRIIB-Fc on body mass of mice exposed oropharyngeally to either asbestos or silica dust. E1 and E2 show time points of dust exposure. **B**. Effect of activin inhibition on skeletal muscle mass. In asbestos-exposed animals, 5 animals with equal starting mass were chosen for measurement. In silica-exposed animals N = 9 and in controls N = 6. **C**. Epididymal and retroperitoneal fat masses of mice treated with sActRIIB-Fc and exposed to either asbestos (Asb, A; N = 5/group) or silica (B; N = 9 in the exposed animal groups and N = 5 in the control (Ctrl) group).

As marked upregulation of activin subunits was observed both in human and mouse IPF tissue, we explored the effects of inhibition of activin function on inflammation, fibrosis, and body composition using a soluble inhibitor comprised of the extracellular portion of the ActRIIB fused to the Fc portion of human IgG. Treatment with sActRIIB-Fc started immediately after exposure of mice either to asbestos or silica (day 1). After exposure, there was a transient reduction or slowing down in the weight gain of the animals (Figure 4A). After two weeks, the mice treated with sActRIIB-Fc showed a clear increase in body weight when compared to both non-treated and non-treated, non-exposed controls. By the end of the follow-up time, the sActRIIB-Fc treated mice were approximately 4 g heavier than the non-treated, exposed control animals in both models. Since sActRIIB-Fc is known to block myostatin and activin-A function as an inhibitor of muscle growth [17, 20, 21] weight gain was expected. Measurements of quadriceps femoris muscle and gastrocnemius muscles (Figure 4B) as well as extensor digitorum longus, soleus and tibialis anterior muscle mass (not shown) showed significant increase in sActRIIB-Fc-treated animals, while no alterations were observed in non-treated, exposed animals (Figure 4B). On average, muscle masses were increased by 35.9% (asbestos-exposed) and 30.7% (silica-exposed) in the sActRIIB-Fc-treated animals. Heart mass was comparable in all the animal groups (not shown). These results confirm the expected function of the soluble inhibitor in both models and provide evidence that exposure of mice to significant amounts of silica or asbestos do not lead to permanent muscle atrophy.

Epididymal fat mass was measured in all the animal groups and retroperitoneal fat mass in the silica model. In mice exposed to asbestos, epididymal fat mass decreased (Figure 4C) in both the treated and non-treated groups. In silica-exposed mice, epididymal fat mass decreased marginally with a further decrease in the sActRIIB-Fc-treated mice (Figure 4C). Furthermore, retroperitoneal fat mass decreased in both silica-exposed animal groups (not shown). These results suggest that fibrotic mice have a reduced fat mass, which may reflect increased energy consumption due to either inflammation or increased respiratory work.

### Effect of activin neutralization on BALF cellular composition and tissue inflammation in fibrotic mice

**Figure 5:**
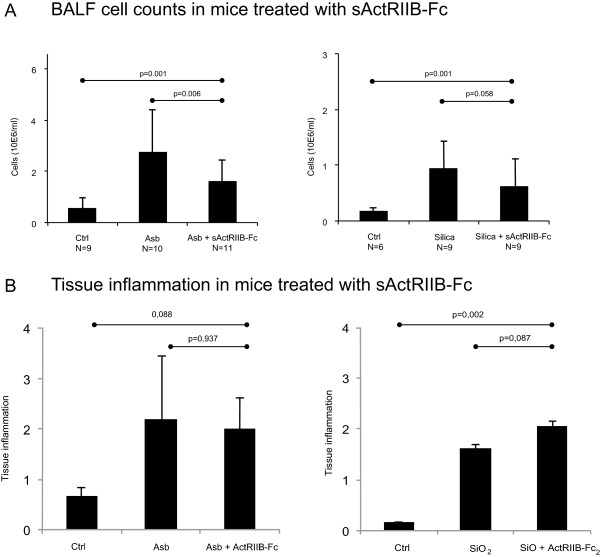
**Effect of activin inhibition to lung inflammation in experimental fibrosis.** **A**. Effect of activin inhibition using sActRIIB-Fc on bronchoalveolar lavage fluid total cell numbers in asbestos (Asb)- and silica-exposed mice. **B**. Effect of sActRIIB-Fc on inflammatory cell score from paraffin sections of these mice (tissue inflammation). *P* values from Kruskal-Wallis (comparing three groups) and MannWhitney U test (comparing two groups).

BALF total cell counts increased in mice exposed to asbestos or silica as expected (Figure 5A). In both models, there was a clear decrease of alveolar total cell numbers in the animals treated with the inhibitor, suggesting that neutralization of activins has an attenuating effect on the cellular response to alveolar injury. The relative numbers (percentages) of neutrophils and lymphocytes increased significantly in asbestos-exposed mice, and this was not altered by treatment with sActRIIB-Fc (not shown). There were no significant changes in tissue inflammatory cell numbers (Figure 5B).

### Effect of activin neutralization on lung profibrotic gene expression and fibrosis in two different mouse fibrosis models

**Figure 6:**
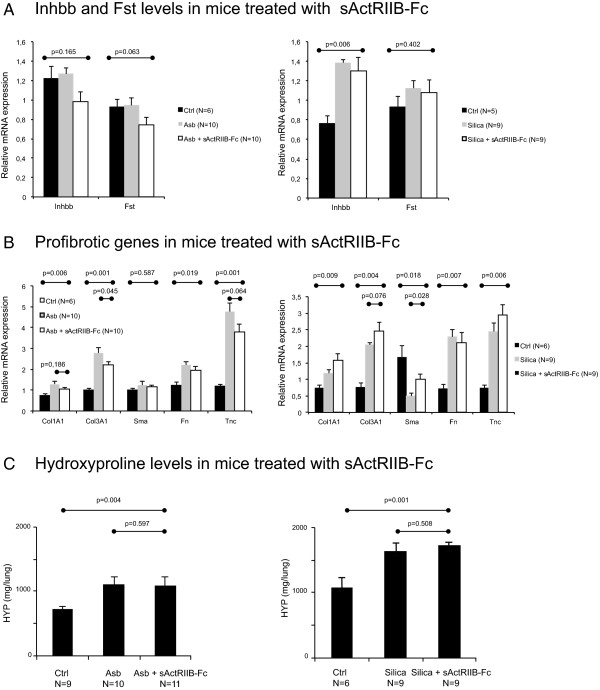
**Effect of activin inhibition by sActRIIB-Fc in asbestos (Asb)- and silica-exposed mice (A)**. Fibrosis-related genes collagen (Col1A1, Col3A1), smooth muscle actin (Sma), fibronectin (Fn), and tenascin-C (Tnc) relative mRNA levels are shown in **B**. Lung total hydroxyproline (HYP) levels **(C)** did not show any significant alterations between groups treated with sActRIIB-Fc and the non-treated control. *P* values from Kruskal-Wallis (comparing three groups) and Mann-Whitney U test (comparing two groups) are indicated in the figure.

**Figure 7:**
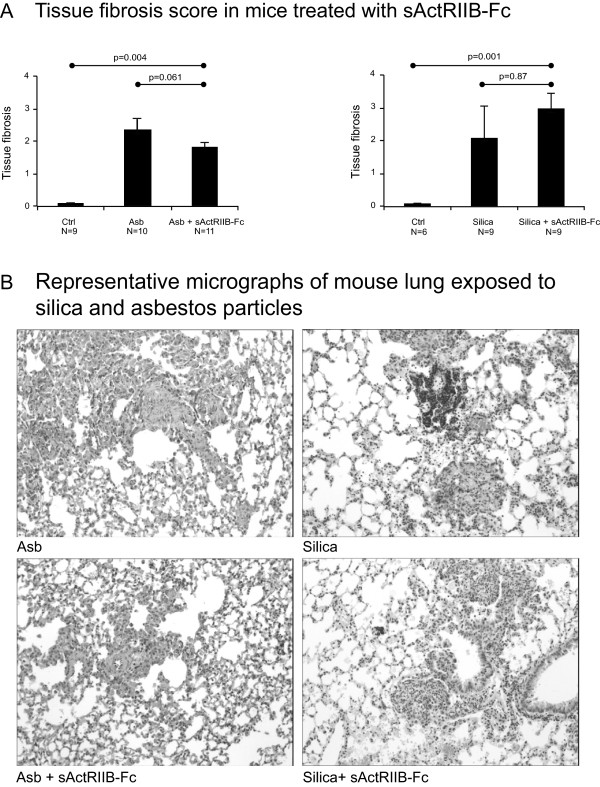
**Effect of activin inhibition to fibrotic alterations in experimental fibrosis.** The histological fibrotic alterations related to asbestos (Asb) and silica exposure, and the effect of sActRIIB-Fc to these alterations in mice **(A)**. The photomicrographs **(B)** show representative haematoxylin-eosin-stained sections from the treated and non-treated groups in both the asbestos- and silica-exposed groups. P values from Kruskal-Wallis (comparing three groups) and Mann-Whitney U test (comparing two groups) are indicated in **A**.

As the upregulation of activins was observed in human lung tissue in relationship to lung fibrosis, we wanted to test whether efficient blockade of activins could modulate the fibrotic response in these two different mouse models of progressive lung fibrosis. Lung tissue mRNA expression levels of *Inhba* were not altered by treatment with sActRIIB-Fc (not shown). There was a slight, but statistically nonsignificant, decrease in *Inhbb* expression in sActRIIB-Fc-treated asbestos-exposed mice (Figure 6A). In agreement with the known regulatory effect of activin on follistatin expression [26], treatment with the sActRIIB-Fc decreased *Follistatin* mRNA expression in the lung tissue of these mice (Figure 6A). Interestingly, there was also a trend towards decreased mRNA expression of major fibrotic genes (*Col1A1*, *Col3A1*, and *tenascin-C*) in sActRIIB-Fc treated asbestos-exposed animals (Figure 6B). However, these alterations were not reflected in tissue hydroxyproline levels (Figure 6C), but there was a 20% decrease in the histological fibrosis score evaluated from haematoxylin-eosin-stained lung tissue sections in the asbestos-exposed sActRIIB-Fc treated animals in comparison to controls (Figure 7). In contrast to what was observed in the asbestos-exposed animals, there was no reduction of lung tissue *Follistatin* mRNA expression in silica-exposed, sActRIIB-Fc-treated mice (Figure 6A). Expression of fibrotic genes and histological fibrosis score tended to further increase slightly in the treated animals (Figures 6B,D). This suggests that the mechanism of fibrosis formation in these two mouse models is different. Hydroxyproline levels were unaltered by sActRIIB-Fc (Figure 6C).

## Discussion

TGF-ß family members are key regulators in a number of fibrotic tissue processes. TGF-ß1 is the major pro-fibrotic isoform associated with the pathogenesis of IPF, and high levels of the signal transducer protein P-Smad2/3 are found in the hyperplastic epithelium and throughout the lung parenchyma [23]. BMPs counterbalance TGF-ß-mediated processes and induce signaling mainly through P-Smad1/5/8. Downregulation of BMP-6 is associated with increased renal fibrogenesis in mice [27], the BMP inhibitor protein gremlin is highly upregulated in the IPF lung [28], and the therapeutic administration of BMP-7 in a number of different experimental models reverses fibrotic alterations [29–31]. Increased activin-A levels are associated with acute respiratory injury [12]. However, the role of activin isoforms in lung fibrosis is less well characterized. Activins signal mainly through the P-Smad2/3 pathway, but recent reports suggest also activation of the P-Smad1/5/8 pathway by activin-B [9]. In this study, activin-B was highly expressed in the human and mouse fibrotic lung, and follistatin was upregulated in the human IPF lung. Previously, upregulation of activin-A has been reported in human autopsy specimens [13] and in mice after bleomycin challenge [15].

To study possible antifibrotic effects *in vivo*, we used two different models of progressive fibrosis where activin subunits were expressed differently (in the asbestos model only activin-A was upregulated, whereas in the silica model both activin-A and -B were upregulated). In asbestos-exposed mice *Inhba* mRNA levels were elevated, and blocking activin-A activity with administration of sActRIIB-Fc reduced BALF cell counts; a trend towards reduced expression of fibrotic genes such as *Col1A1*, *Col3A1* and *tenascin-C* was also observed. Furthermore, tissue fibrosis score measured from haematoxylin-eosin-stained tissue sections indicated a 20% decrease in fibrotic alterations. sActRIIB-Fc reduced *Follistatin* mRNA expression in asbestos-exposed animals, indicating possible functional neutralization of activin-A in lung tissue. The hydroxyproline level, which is usually used to measure the effects of antifibrotic medication in experimental models, was not reduced. In silica-exposed mice, we were unable to show any effects on fibrosis, except for a slight increase in histopathological alterations. Taken together, these results indicate that the neutralization of activins is not sufficient to extensively reduce fibrosis, despite small histological alterations in the treated mice. We did find a useful correlation of collagen and tenascin mRNA levels with the observed histopathologial alterations: there was a trend towards increased expression of fibrotic genes in parallel with increased tissue fibrosis score in the sActRIIB-Fc-treated silica-exposed mice and, vice versa, with increased fibrotic changes these genes were upregulated. These results indicate that more sensitive assays than hydroxyproline measurements could be useful in the quantification of fibrosis in mouse models.

Localization of activin subunits was possible in human but not in mouse tissue due to lack of suitable antibodies. Specific immunoreactivity for activin-A and -B was observed in the hyperplastic alveolar epithelium, a hallmark pathological finding of human IPF/UIP histopathology. Loss of epithelial cell integrity has been recognized as a key process in pulmonary fibrosis [32]. There is a need for specific disease markers, and activin-B can be considered to be a potential marker of alveolar epithelial cell activation. The neutralization of activins by sActRIIB-Fc was not sufficient to convincingly reduce fibrosis in two separate mouse models of pulmonary fibrosis. However, the inhibition of activin activity was found to contribute to the alveolar cellular response to particulate exposure without directly affecting inflammatory cell numbers (alveolar and tissue infiltrating neutrophils and lymphocytes). This result supports the previous finding that sActRIIB-Fc treatment may be beneficial in the reduction of acute alveolar injury [12]. In itself, an injury-decreasing compound would be welcome in clinical use as a potential therapeutic molecule for acute IPF exacerbation, an often-fatal condition that affects up to 15% of IPF patients [33]. So far, no clinical trials have had consistent effects on IPF exacerbation [2–4]. If further developed for clinical use, activin inhibition could be considered as a candidate rescue therapy for acute IPF exacerbation.

Our results indicate that the use of multiple experimental models in therapeutic studies is crucial. The induction of the expression of activin subunits was different in the two models used for activin neutralization experiments, which is likely to affect the outcome. Strong induction of activin-B, similar to human IPF patient samples, associated with silica-induced fibrosis. Activin-B localization in human activated epithelium suggests pathologic function; however, the recent identification of the ALK-3/P-Smad1/5/8 pathway as an alternative mediator of activin-B signals [9] indicates a more complicated signaling system. The use of sActRIIB-Fc, which can sequester in addition to activins also myostatin, nodal, and certain BMP isoforms, notably BMP-4 and BMP-7, may also inhibit positive repair signals [34]. Furthermore, muscle growth induced by inhibition of myostatin function in sActRIIB-Fc-treated animals may contribute to a profibrotic microenvironment. Follistatin treatment has been found to inhibit bleomycin-induced lung inflammation and subsequent fibrosis [16], indicating that blocking activin function can be beneficial.

## Conclusions

We report here an original observation that activin-B and follistatin are upregulated in the human IPF lung, and that activin-A, -B and follistatin protein localize to the activated, pathologic alveolar epithelium in the human IPF/UIP lung. A high level of upregulation and disease-specific localization of activin-B in the IPF lung may indicate that activin-B could have potential as a biochemical marker for IPF progression. Epithelial cell activation in IPF is currently considered as a hallmark of the pathogenesis of IPF. Endoplasmic reticulum (ER) stress, the reinduction of developmental genes such as the BMP and Wnt pathway genes are key elements that distinguish the IPF lung from the healthy adult lung. In this study, we show marked upregulation of yet another set of developmental genes, activin-A, -B and follistatin, which are almost completely absent from the healthy lung. Our findings by using specific blocking indicate that activins are not directly linked to disease progression or fibrosis generation but show a clear reduction in the alveolar response to injury in two mouse models of particulate exposure. Finally we postulate, that activin-B as a soluble protein can have potential as an IPF biomarker.

## Abbreviations

ALK: Activin-like kinase
BALF: Bronchoalveolar lavage fluid
BMP: Bone morphogenetic protein
COL: Collagen
FST: Follistatin
INH: Inhibin
IPF: Idiopathic pulmonary fibrosis
TGF-ß: Transforming growth factor-ß
UIP: Usual interstitial pneumonia.
